# CXXC5 (Retinoid-Inducible Nuclear Factor, RINF) is a Potential Therapeutic Target in High-Risk Human Acute Myeloid Leukemia

**DOI:** 10.18632/oncotarget.1195

**Published:** 2013-08-15

**Authors:** Audrey Astori, Hanne Fredly, Thomas Aquinas Aloysius, Lars Bullinger, Véronique Mansat-De Mas, Pierre de la Grange, François Delhommeau, Karen Marie Hagen, Christian Récher, Isabelle Dusanter-Fourt, Stian Knappskog, Johan Richard Lillehaug, Frédéric Pendino, Øystein Bruserudg

**Affiliations:** ^1^ Inserm, U1016, Institut Cochin, F-75014, Paris, France; ^2^ CNRS, UMR8104, F-75014, Paris, France; ^3^ Université Paris Descartes, Sorbonne Paris Cité, Paris, France; ^4^ Section for Hematology, Institute of Medicine, University of Bergen, Norway; ^5^ Department of Medicine, Haukeland University Hospital; Bergen, Norway; ^6^ Department of Molecular Biology, University of Bergen, Bergen, Norway; ^7^ Department of Internal Medicine III, University of Ulm, Ulm, Germany; ^8^ Inserm, Unité Mixte de Recherche 1037-Cancer Research Center of Toulouse, CNRS 5294, Université de Toulouse, Centre Hospitalier Universitaire Purpan, F-31059, Toulouse, France; ^9^ Service d'Hématologie, Centre Hospitalier Universitaire Purpan, H&ocirc;pital Purpan, F-31059, Toulouse, France; ^10^ GenoSplice, Hôpital Saint-Louis, F-75010, Paris, France; ^11^ UPMC, Pierre and Marie Curie University, GRC n°07, Groupe de Recherche Clinique sur les Myéloproliférations Aiguës et Chroniques MyPAC, F-75012, Paris, France; ^12^ AP-HP, Hôpital Saint-Antoine, Service d'Hématologie et Immunologie Biologiques, F-75012, Paris, France

**Keywords:** Acute myeloid leukemia, CXXC5/RINF, chemotherapy, apoptosis

## Abstract

The retinoid-responsive gene CXXC5 localizes to the 5q31.2 chromosomal region and encodes a retinoid-inducible nuclear factor (RINF) that seems important during normal myelopoiesis. We investigated CXXC5/RINF expression in primary human acute myeloid leukemia (AML) cells derived from 594 patients, and a wide variation in CXXC5/RINF mRNA levels was observed both in the immature leukemic myeloblasts and in immature acute lymphoblastic leukemia cells. Furthermore, patients with low-risk cytogenetic abnormalities showed significantly lower levels compared to patients with high-risk abnormalities, and high RINF/CXXC5/ mRNA levels were associated with decreased overall survival for patients receiving intensive chemotherapy for newly diagnosed AML. This association with prognosis was seen both when investigating (i) an unselected patient population as well as for patients with (ii) normal cytogenetic and (iii) core-binding factor AML. CXXC5/RINF knockdown in AML cell lines caused increased susceptibility to chemotherapy-induced apoptosis, and regulation of apoptosis also seemed to differ between primary human AML cells with high and low RINF expression. The association with adverse prognosis together with the antiapoptotic effect of CXXC5/RINF suggests that targeting of CXXC5/RINF should be considered as a possible therapeutic strategy, especially in high-risk patients who show increased expression in AML cells compared with normal hematopoietic cells.

## INTRODUCTION

We have recently identified a novel retinoid-responsive gene (CXXC5) that encodes a retinoid-inducible nuclear factor (RINF), and expression studies as well as gene silencing experiments suggest that RINF is important in normal myelopoiesis [1]. The RINF gene localizes to the 5q31.2 chromosomal region that can be involved in chromosomal abnormalities associated with various myeloid malignancies, including the low-risk 5q-variant of human myelodysplastic syndromes (MDS) [1, 2] and the high-risk del5 and −5 abnormalities in human acute myeloid leukemia (AML) [3].

AML is an aggressive malignancy characterized by bone marrow infiltration of immature leukemic myeloblasts [3, 4]. However, it is a very heterogeneous disease both with regard to leukemogenesis (i.e. AML-associated genetic abnormalities) as well as the chemosensitivity and thereby the risk of relapse [3, 5]. On the other hand, RINF seems to be an important early regulator of normal myelopoiesis [1]. In the present study we therefore investigated the expression of RINF in non-promyelocytic variants of AML. We observed a wide variation of RINF expression in primary human AML cells and an association between high expression and decreased overall survival, and RINF seems to mediate antiapoptotic effects. Taken together, these observations suggest that RINF should be considered as a possible therapeutic target in human AML, but whether RINF expression in addition is an independent prognostic parameter cannot be judged from the present data and will require further clinical studies.

## RESULTS

### CXXC5/RINF expression by primary human AML cells shows a wide variation

**Figure 1:**
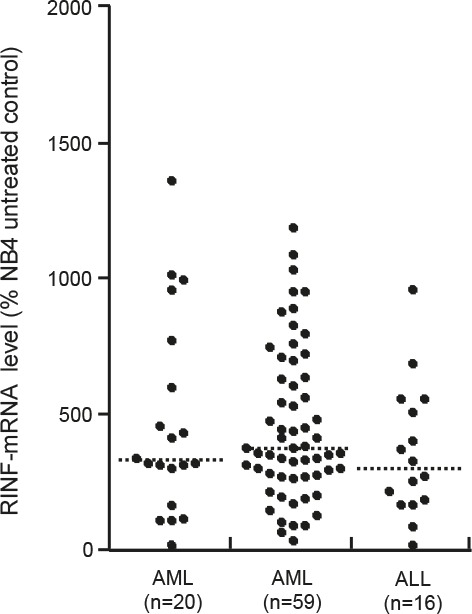
RINF expression by primary human acute leukemia cells RINF expression was determined for primary AML cells derived from the bone marrow of 20 French patients (LEFT); primary AML cells derived from the peripheral blood of 59 Norwegian patients (MIDDLE); and for primary ALL cells derived from the blood of 16 patients (RIGHT). The median RINF level is indicated in the figure.

We analyzed RINF expression for 59 unselected Norwegian AML patients (Table 1). The expression showed a wide variation between patients (Fig. 1) without any significant correlations to age or gender. Samples derived from patients with *de novo* AML showed a slight but significant decrease of the RINF levels compared to patients with relapsed or secondary AML (Mann-Whitney's test, p<0.05). A similar median level and variation range was also seen for ALL blasts derived from 14 patients (Fig. 1).

**Table 1:** Clinical and biological characteristics of 59 Norwegian AML patients included in the study

Age (median, range)	62.5 years (range 27-88)
	
Number of patients with: Secondary AML AML relapse de novo AML	15 14 30
	
FAB classification (number of patients) M0/M1 M2 M4/M5	24 12 23
	
Expression of CD34 (percentage of patients)*	36%
	
Cytogenetic abnormalities (patient numbers)* Normal Good Intermediate Adverse Not determined	23 4 7 11 12
	
Frequency of Flt3- internal tandem duplication (ITD)	36%
	
Frequency of NPM1 mutations	44%
	

* CD34 positivity was defined as at least 20% of the cells staining positive for the CD34 stem cell marker. Cytogenetic abnormalities were classified according to the MRC guidelines. Cytogenetic analysis was not available for 12 patients; this was due to either no mitoses to analyze, death or start of chemotherapy in elderly patients due to critical illness, hyperleukocytosis or other severe complications before sampling for cytogenetic analysis was possible. Cytogenetic analyses were only performed on freshly isolated cells and never on cryopreserved cells [13].

The French-American-British (FAB) classification was used to compare RINF expression for AML cells with minimal differentiation (FAB-M0/M1; median level 517, variation range 101-950) with neutrophil (FAB-M2; median 474 and range 193-1477) or monocytic differentiation (FAB-M4/M5; median 334 and range 29-951). Neutrophil differentiation was not associated with altered RINF expression, whereas monocytic differentiation was associated with decreased RINF mRNA levels (Mann-Whitney test, p=0.0472) compared with AML cells showing minimal differentiation. Finally, we did not observe any association between RINF expression and surface expression of the CD34 stem cell marker.

We compared RINF expression for patients with low- (median 243, variation range 87-357), intermediate- (794, 29-1189), high-risk (434, 29-951) and normal cytogenetics (404, 89-1289). The difference between high- and low-risk cytogenetics then reached a borderline significance (Mann-Whitney's test, p=0.0475), whereas no statistically significant differences were detected when comparing the other groups. Finally, the presence of high-risk Flt3 internal tandem duplications (Flt3-ITD) or low-risk Nucleophosmin (NPM) −1 mutations showed no associations with RINF expression in this Norwegian cohort (data not shown), whereas analysis by Q-RT-PCR in 40 unselected patients showed a significant correlation between RINF and WT1 expression (Spearman's correlation test, ρ=0.661, p=0.00001).

We also analyzed the French AML patient cohorts; highly enriched AML blasts were then derived from the bone marrow of 20 patients. Analysis of these patients confirmed that RINF expression shows a wide variation in primary human AML cells and this was similar to AML blasts derived from peripheral blood both when compared with the Norwegian patients (Fig. 1) and 20 French patients (median RINF expression 369).

### CXXC5/RINF is expressed by normal hematopoietic cells

We investigated RINF expression in normal mononuclear bone marrow cells derived from 12 healthy individuals (bone marrow mononuclear cells) and in CD34^+^ bone marrow cells (n=11). The median expression in these bone marrow cells were 152.2 and 497, respectively.

### Mutations of the CXXC5/RINF gene are uncommon in AML

We first investigated *RINF* gene status in primary AML samples from 43 unselected Norwegian patients (Table 1). For these patients no mutation was detected in the coding region of the *RINF* gene when we performed a complete sequence analysis of the Coding DNA Sequence (data not shown). Two silent Single Nucleotide Polymorphisms rs3756677(C>T) and rs356445 (G>A), were found in exon 3 of the *RINF* gene for 3 patients. The first SNP is located in the 5'UTR while the second is a synonymous SNP (Ala126>Ala126) located in the open reading frame. These SNPs were consistently associated (3/3). The allelic frequency of the double silent SNP (3/43, 6.9%) was not statistically different from the one observed in the European population (~4 %, data not shown).

### CXXC5/RINF expression in primary human AML cells is associated with survival

**Figure 2:**
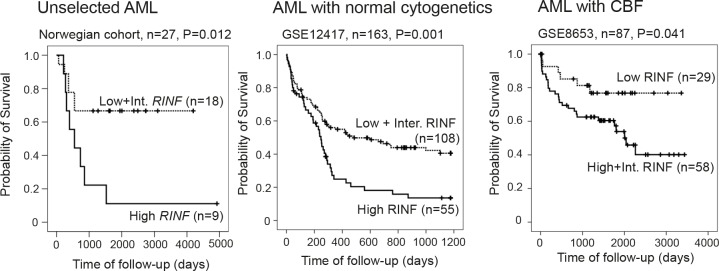
High *RINF* mRNA expression is associated with decreased overall survival in AML; an analysis of three different patient populations The Kaplan-Meier curves (for survival analysis) and the log-rank test were performed by using the statistical SPSS 19.0. *P* values (log-rank test) of the comparison of the various groups of patients are indicated in each of the figures. (LEFT) The figure shows the results for the 27 unselected patients with newly diagnosed AML (Norwegian cohort) who received intensive chemotherapy. The figure compares the survival for the 9 patients with the highest RINF levels with the 18 patients with intermediate and low expression. The survival differed significantly between the two groups (p=0.012). (MIDDLE) The microarray dataset (Affymetrix GeneChip Human Genome HG-U133B) performed by Metzeler KH *et al*. [6] (163 patients) was downloaded from the Gene Expression Omnibus website (http://www.ncbi.nlm.nih.gov/geo/) with accession number GSE12417. The whole raw data were normalized using RMA (Robust Multiarray Averaging method) with the Expression Console software from Affymetrix. Since there were 3 probesets targeting *CXXC5* (222996_s_at, 224516_s_at and 233955_x_at), the number of variable was reduced by PCA reduction analysis to determine the *CXXC5* mRNA expression. The patients have been classified in 3 equivalent groups according to a high (n=55), an intermediate (n=54), or a low (n=54) *RINF* expression level. Here, the low (n=54) and intermediate (n=54) groups have been fused because they had similar survival. Note that because of an odd number of patients (n=163) the groups included different numbers of patients (55 *versus* 54). (RIGHT) RINF expression and survival was compared for 87 patients with core-binding factor AML; this analysis was also based on public microarray data [7] and again we observed a significant association between overall survival and RINF mRNA expression

We investigated a cohort of consecutive Norwegian patients including relapse patients (Table 1); the median age was relatively high (64 years) (Table 1) and several patients were therefore regarded as unfit for intensive chemotherapy. For these reasons, only 27 of these patients received intensive induction chemotherapy with cytarabine plus an anthracycline followed by 3 or 4 consolidation cycles with intensive chemotherapy for newly diagnosed leukemia (Fig. 2; Supplementary Fig. 1 and Supplementary Table 1). The 9 patients with the highest RINF expression in the AML cells then showed a significantly decreased overall survival compared with the groups with intermediate or low RINF levels (p=0.012). A significant association between overall survival after chemotherapy and RINF expression in the marrow-derived AML cells was also observed for the 20 French patients (Supplementary Fig. 2 and Supplementary Table 2; p=0.037).

We analyzed the association between RINF expression and overall survival for patients included in three different clinical studies. Firstly, the study by Metzeler KH *et al*. [6] included 163 patients with normal cytogenetics and again we observed a significant association between RINF expression and survival; high levels were associated with an adverse prognosis and decreased overall survival (Fig. 2). Secondly, we analyzed 87 patients with core-binding factor AML published by Bullinger L et al. [7] and we observed significant associations between overall survival and RINF expression both when analyzing the overall results (Fig. 2) and when patients with inv(16) and t(8;21) were analyzed separately. In contrast to the two previous cohorts patients with intermediate levels showed a survival curve close to the patients with high levels, and for this reason they were analyzed together. The association was strongest for inv(16) (data not shown). Finally, an association between RINF level and survival was also seen for the study by Gaidzik VI et al. [8]. This last study included mainly patients with inv(16) (31 patients) and for these patients we observed that (i) patients with high and intermediate RINF expression showed a similar survival; and (ii) low RINF expression was associated with a significantly increased overall survival compared with intermediate/ high patients (p=0.026). Thus, analyses of five different patient populations all show that high RINF expression is associated with decreased overall survival.

### CXXC5/RINF silencing sensitizes AML cells to chemotherapy

**Figure 3:**
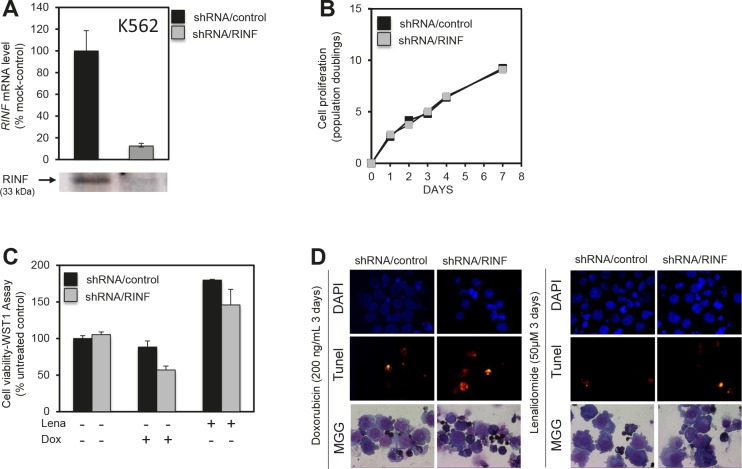
RINF knock-down sensitizes K562 cells to doxorubicin- and lenalidomid-induced apoptosis K562 cells stably expressing shRNA/RINF or a control shRNA/scramble were examined. (A) RINF expression; shRNA/RINF was associated with decreased RINF expression both at the mRNA and protein level. (B) Effect of RINF knockdown on AML cell proliferation, RINF knockdown did not alter K562 proliferation during 7 days of *in vitro* culture. (C, D) K562 cells were cultured for 4 days with 50 μM of lenalidomide or 200 ng/μl of doxorubicin. Cell viability and cell death was then assessed at the same time. (C) WST1-cell viability and proliferation assay or (D) TUNEL assay was realized before a DAPI staining. (D) Morphology was evaluated by light microscopy of May-Grünwald-Giemsa stained cytospin smears. The observations are consistent with an increased susceptibility to chemotherapy-induced apoptosis after RINF knockdown.

To further investigate whether RINF contributes to the chemoresistant phenotype of high-risk patients we investigated the effect of RINF knockdown on the susceptibility of five different immature myeloid leukemic cell lines to chemotherapy-induced apoptosis, including K562, MV4-11, OCI-AML3, NB4 and HL60. The highest RINF mRNA levels were observed for K562 and MV4-11 cells, and these cells showed an increased susceptibility to drug-induced apoptosis after RINF silencing. The results for K562 are presented in detail in Fig. 3. An efficient knock-down of RINF at the protein level was documented (Fig. 3A), but the *in vitro* proliferation was not altered (Fig. 3B) and apoptosis was minimal during *in vitro* culture in the absence of chemotherapy (Fig. 3C, left). However, RINF knockdown was associated with increased sensitivity to daunorubicin- and lenalidomide-induced apoptosis (Figs. 3C and 3D). The susceptibility to drug-induced apoptosis was not affected by RINF silencing for the three cell lines with low expression (data not shown). All these effects were reproduced in independent experiments.

### Low CXXC5/RINF expression is associated with decreased viability of in vitro cultured primary AML cells but does not have a major impact on leukemic cell proliferation

**Figure 4:**
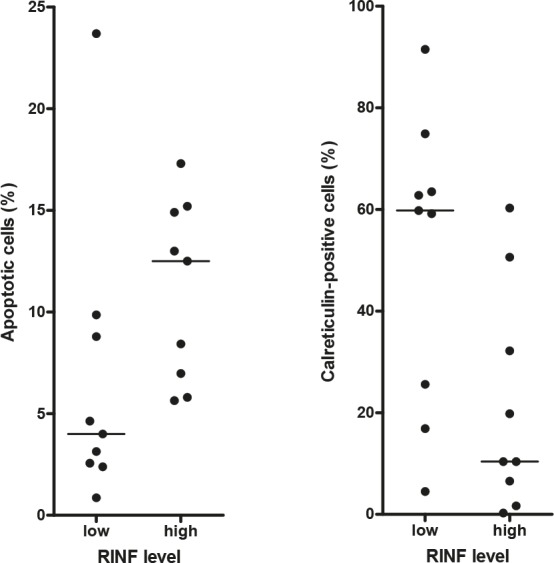
Stress-induced spontaneous *in vitro* apoptosis and lenalidomide-induced apoptosis in primary human AML cells, a comparison of patients with high and low RINF expression Primary human AML cells were derived from 9 patients with low and 9 patients with high RINF expression. (LEFT) Primary AML cells with low RINF expression were mainly necrotic after 18 hours *in vitro* culture in medium alone, and the leukemic cells from these patients showed a low level of viable as well as apoptotic (see the figure for lenalidomide) cells both in cultures prepared in medium alone and cultures with lenalidomide. In contrast, for patients with high RINF expression a higher viability was seen after culture in medium alone. However, as shown in the figure an increased fraction of apoptotic cells was then detected for high-RINF patients when cells were cultured in the presence of lenalidomide compared with the corresponding cultures for low-RINF patients (see the figure; Mann Whitney U-test, p=0.0004), and at the same time the viability further decreased and the fraction of necrotic cells increased for the high-RINF patients. Thus, maximal viability reduction is achieved by spontaneous apoptosis alone for low-RINF patients but for high-RINF patients combined spontaneous and lenalidomide-induced apoptosis is required to achieve the maximal reduction. (RIGHT) The calreticulin exposure of AML cells cultured with lenalidomide 0.1 μM was determined by flow cytometry [11]. A dye-exclusion Dead/Live Viability/Vitality analysis was used to separate the leukemic cells into these two major subsets, and calreticulin expression could thereby be analyzed separately for dead and viable cells. The figure compares calreticulin exposure after culture with lenalidomide (mean fluorescence intensity, MFI) for the dead cells derived from patients with high and low RINF expression (Mann Whitney U-test, p=0.035). The two patient groups showed no difference in calreticulin exposure when analyzing viable cells, and there was no difference after culture in medium alone either (data not shown)

We compared apoptosis induction during *in vitro* culture for the 9 patients with the highest and the 9 patients with the lowest RINF expression in the Norwegian cohort (see Fig. 1). The leukemic cells were cultured for 18 hours before the frequencies of viable, apoptotic and necrotic cells were determined. *In vitro* cultured primary AML cells show spontaneous or stress-induced apoptosis that varies considerably between patients [9, 10]; this was also true both for the AML cells with low (median viability 22.5%, range 1.1-66.6%) and high (median viability 44.2%, range 9.6-56.2%) RINF expression. The viability in drug-free cultures was also compared with cells cultured with lenalidomide 0.1, 0.5, 1.0 μM. For cells with low RINF expression, there was a low viability after culture in medium alone and the viability seemed to have reached a plateau and was not further decreased by lenalidomide (lenalidomide 1.0 μM; median viability 15.5%, range 1.4-53.9%). In contrast, for AML cells with high RINF expression, the viability after culture in medium alone was higher and lenalidomide caused a statistically significant and dose-dependent further decrease in viability (lenalidomide 0.1 μM, no significant effect; lenalidomide 0.5 μM, median viability 31.6%, range 7.0-57.3%, p=0.0098; lenalidomide 1.0 μM, median viability 31.6, range 6.8-50.8; p=0.0078) compared with the corresponding drug-free cultures (median viability 44,2%, see above). The low-RINF patients showed a similar low level of viable and apoptotic cells both after culture in medium alone and in the presence of lenalidomide, whereas the lenalidomide-induced decrease in viability for the high-RINF patients was associated with an increased fraction of apoptotic cells compared with the corresponding cultures for low-RINF patients (Fig. 4; Mann-Whitney U-test, p=0.0004). To conclude, low-RINF patients achieved a maximal decrease in viability by spontaneous *in vitro* apoptosis alone, whereas for high-RINF patients the maximal decrease in viability required combined spontaneous and lenalidomide-induced apoptosis.

We also compared the surface exposure of calreticulin for primary AML cells derived from the same patients with high and low RINF expression (see Fig. 1); the exposure was then compared after culture in medium alone and in the presence of lenalidomide 0.1 μM (Fig. 4). The surface exposure of calreticulin by non-viable AML cells differed significantly when comparing patients with high and low RINF expression (Fig. 4; Mann Whitney U-test, p=0.035), but this difference was only seen when cells were cultured with lenalidomide.

Taken together our studies of AML cell apoptosis support the hypothesis that RINF is important for regulation of viability/apoptosis also in primary human AML cells, and the lenalidomide studies further suggest that the chemotherapy-induced apoptotic phenotype differs between patients with high and low RINF expression.

In a recent publication we describe that AML patients can be subclassified into three major subsets based on their *in vitro* proliferative capacity: (i) patients with spontaneous/autocrine and strong cytokine-dependent proliferation in the presence of several growth factors; (ii) patients only showing cytokine-dependent proliferation; and (iii) patients showing no or very weak autocrine and cytokine-dependent proliferation (13). Patients with high and low RINF expression showed a similar distribution within all three subsets (26 unselected patients examined, variation range for RINF 28-951, data not shown).

### DISCUSSION

The CXXC5/RINF gene localizes to the 5q31.2 chromosomal region that can be involved in chromosomal deletions associated with AML, and for this reason we investigated the possible role of RINF in this disease. RINF expression was investigated in primary human AML cells derived from a large group of unselected patients. We did not detect mutations in the RINF encoding regions and in this relatively small Norwegian cohort there were no significant associations between RINF mRNA expression and FLT3-ITD or NPM1 mutations. Importantly, high RINF expression was associated with an adverse prognosis and experimental studies demonstrated that RINF mediated antiapoptotic activity; taken together our observations therefore suggest that therapeutic targeting/ inhibition of RINF should be considered for the treatment of high-risk AML. Whether RINF expression in addition is an independent prognostic parameter in human AML requires additional clinical studies and cannot be judged from our present results.

Our methodological strategy for sampling of AML cells from patients with relatively high levels of circulating AML cells has been described and discussed in detail previously [12], and our previous data have shown that the patients are representative for AML in general with regard to the major prognostic parameters, i.e. clinical chemosensitivity [12-14]. By using this strategy, highly enriched AML cell populations could be prepared by gradient separation alone; more extensive cell separation procedures may alter the molecular profile or functional characteristics of primary AML cells [14]. However, despite these previous characterizations, we emphasize that our results may be representative only for the selected subset of patients, even though our patients showed an expected distribution with regard to morphological signs of differentiation as well as genetic abnormalities (Table 1).

We observed an association between monocytic differentiation and RINF expression, and this is similar to normal hematopoiesis where RINF expression also depends on the differentiation status of myeloid cells [1]. We also observed a lower level for patients with favorable compared to adverse cytogenetic abnormalities in our Norwegian cohort, but this last observation has to be interpreted with great care because we examined unselected patients with a high median age and very few patients with favorable cytogenetics were therefore included [12]. Finally, we could not detect mutations of the CXXC5 gene for any of our patients, and the same was also observed in a previous study [15].

We investigated the association between RINF expression and overall survival for five different patient populations receiving intensive chemotherapy for newly diagnosed AML. The overall survival for these relatively young patients is mainly reflected by the clinical chemosensitivity, ie. the frequencies of primary resistant disease and later AML relapse [3]. A similar association between high RINF levels and adverse prognosis was observed both for (i) unselected patients; (ii) patients with normal cytogenetics and (iii) patients with genetic abnormalities affecting core-binding factors who have a good prognosis (Fig. 2). These observations support the hypothesis that high RINF expression is associated with an adverse prognosis. A similar association has been observed in patients with breast cancer where high expression is also associated with adverse prognosis [16].

We used well-characterized AML cell lines to study the effects of RINF expression on chemosensitivity. K562 cells then represents a standardized experimental model of immature myeloid leukemic cells. Our experiments demonstrated that RINF knock-down was associated with an increased susceptibility to chemotherapy-induced apoptosis. This observation suggests that RINF expression contributes to the chemoresistant AML cell phenotype.

We compared the viability of primary AML cells during *in vitro* culture for patients showing high and low RINF expression. Both groups showed an expected spontaneous *in vitro* apoptosis and a reduction of viability during culture [9, 10]. The cells were also cultured without and with lenalidomide; this drug was chosen because it is now tried in AML therapy [17-20] and it can be used in the treatment of MDS with the 5q- abnormality and low RINF expression [1, 2, 21]. In these drug-containing cultures the viability is thus determined by the spontaneous *in vitro* apoptosis together with additional drug-induced apoptosis. A maximal reduction of AML cell viability during *in vitro* culture was reached by stress-induced apoptosis alone for the low-RINF group, whereas the maximal reduction required a combination of stress-induced and drug-induced apoptosis for the high-RINF patients. The two groups also differed in their apoptotic phenotype with regard to calreticulin exposure during cell death. Taken together, these results support the hypothesis that RINF is important for regulation of apoptosis in human AML cells. Similar to the K562 experiments (Fig. 3B) we did not detect any significant correlation between proliferative capacity and RINF expression for primary human AML cells.

Previous studies in other experimental models suggest that pharmacological inhibition of anti-apoptotic intracellular signaling in human AML cells can induce apoptosis or increase proapoptotic effects of chemotherapy [22-26]; therapeutic targeting of CXXC5/RINF may therefore represent a novel and alternative strategy to increase proapoptotic activity and thereby chemosensitivity in human AML cells. The molecular mechanisms behind the proapoptotic activity of CXXC5/RINF silencing in AML cells are not known. Observations in neuronal cells and developing kidney have suggested that WT1 induce RINF expression and thereby downregulate signaling through the WNT-beta-cathenin pathway [27, 28]. The correlation between WT1 and CXXC5/RINF expression may suggest that there is a crosstalk between these two molecules also in primary AML cells. However, the WNT-beta-cathenin pathway can be constitutively activated in AML cells [29] and this activation seems to mediate antiapoptotic effects [30, 31]. Thus, the antiapoptotic effect of RINF in AML cells is probably mediated through other mechanisms than downregulation of proapoptotic signaling through the WNT-beta-cathenin pathway.

RINF was also expressed in normal hematopoietic cells and hematological toxicity is therefore a possibility if RINF inhibition is tried in AML treatment. In our opinion the expression of RINF in normal bone marrow cells does not exclude the possibility to consider RINF targeting in AML therapy. Firstly, preparation of CD34^+^ enriched cells requires more extensive cell separation procedures than simple gradient separation and this *ex vivo* handling may alter RINF expression [13, 14]. Secondly, several patients showed higher AML cell levels than the median levels of normal cells, especially high-risk patients. Thirdly, viability is probably not determined by a single pathway but by the balance between pro- and antiapoptotic signaling; the functional consequences of RINF inhibition will be determined by this balance and may thereby differ between normal and leukemic cells [32]. Finally, as discussed in detail previously combined targeting of different apoptosis-regulating pathways is a promising strategy in cancer treatment [33], and it may then be possible to design combinations that include RINF targeting but at the same time have acceptable hematological toxicity (33). However, the final answer to the important question of toxicity can only come from preclinical pharmacological studies in animal leukemia models [34, 35] and eventually clinical studies.

The importance of stromal cells in carcinogenesis and for chemosensitivity of human malignancies was discussed in a recent review [36]. Targeting of the stromal cells may thus be a possible strategy both in the treatment of solid tumors [37, 38] as well as hematological malignancies [39]. A recent study suggested that targeting of the PI3K-Akt-mTOR pathway will affect bone marrow stromal cells and thereby have indirect effects on AML cells [39]. Future studies should therefore investigate whether CXXC5/RINF is expressed in bone marrow stromal cells and whether CXXC5/RINF targeting will affect stromal cells and thereby have indirect effects on the leukemic cells.

In summary, RINF expression shows a wide variation in primary human AML cells and high levels are associated with decreased overall survival after chemotherapy. Furthermore, RINF seems to mediate antiapoptotic effects. Taken together these observations suggest that therapeutic targeting/inhibition of RINF should be considered as a possible therapeutic strategy in human AML, especially for those patients whose leukemia show a higher expression than normal hematopoietic cells.

## MATERIAL AND METHODS

### Leukemia patients and preparation of leukemic cells

AML and acute lymphoblastic leukemia (ALL) patients (Norwegian cohorts). The study was approved by the local Ethics Committee (Regional Ethics Committee III, University of Bergen, Norway) and samples collected after written informed consent; 59 consecutive patients with high peripheral blood blast counts (>7 × 10^9^/L) were included (Table 1). This selection of patients and the analysis of FLT3 and NPM1 mutations have been described previously [12, 40]. AML cells were isolated by density gradient separation (Lymphoprep, Axis-Shield, Oslo, Norway) and contained at least 95% leukemic blasts. The cells were stored in liquid nitrogen until used in the experiments [12].

AML patients (French cohort). AML samples have been obtained after informed consent and were stored at the HIMIP collection. According to the French law, the HIMIP collection has been declared to the Ministry of Higher Education and Research (DC 2008-307 collection 1) and obtained a transfer agreement (AC 2008-129) after approbation by the “Comité de Protection des Personnes Sud-Ouest et Outremer II” (ethical committee). Clinical and biological annotations of the samples have been declared to the CNIL (Comité National Informatique et Libertés, i.e. Data processing and Liberties National Committee).

### Expression Analysis of RINF Transcript Levels

For the unselected patients cohort from Bergen (n=59), total RNA was purified from snap-frozen cell pellet samples with Trizol (Life Technologies, Inc. Gaithersburg, MD) extraction protocol according to manufacturer's instructions. RNA was dissolved in 100 μl of diethylpyrocarbonate-treated ddH2O after extraction. First-strand complementary DNA synthesis (RT) was carried out starting with total RNA (1 μg) in a 20 μl volume using oligo-dT primers with Transcriptor Reverse Transcriptase (Roche, Basel, Switzerland, Cat. N°03531287001) in accordance with the manufacturer's instructions. Quantitative PCRs (qPCR) were carried out using specific hydrolysis probes targeting *RINF* gene on a LightCycler 480 machine (Roche) in accordance with the manufacturer's instructions of the kit LightCycler® 480 ProbesMaster (Cat. N°04707494001). Relative messenger RNA (mRNA) expressions were normalized to ribosomal protein P2 (*RPLP2*) gene expression in a two-color duplex reaction. Primers and thermocycling conditions are available upon request.

### Sequence analysis of RINF gene

Amplification of the coding region of *RINF* gene was carried out using forward primer 5'-*gtggaccctcggcagttg*-3' and reverse primer 5'-*cacacgagcagtgacattgc*-3'. PCR amplification was carried out using Dynazyme EXT DNA polymerase (FINNZYMES, Espoo, Finland) in a 50 μl reaction mixture containing 1X PCR buffer, 1.5 mM MgCl2, 0.5 mM of each deoxynucleotide triphosphate, 5% dimethyl sulfoxide, and 0.2 μM of each primer and DNA template (0.5 μl cDNA or 1 μl genomic DNA). The PCR conditions were an initial denaturation step of 5 min at 94°C followed by 40 cycles of 30 s at 94°C, 30 s at 63°C, and 1 min at 72°C, followed by a final elongation step of 7 min at 72°C. Before sequencing, PCR products were purified using the ExoSAP-IT kit (GE healthcare, Waukesha, WI, Cat. N° 78201). Sequencing was done using BigDye® v3.1 cycle sequencing kit (ABI, Foster City, CA, Cat. N° 4337456) with specific forward (5'-*gcacaaaagtggtgctgtg*-3') or reverse (5'-*gcgtggtgcaggagcat*-3') sequencing primers in a total volume of 10 μl. Thermal conditions were 25 cycles of denaturation at 96°C for 10 s, annealing at 50°C for 5 s, and elongation at 60°C for 4 min. Capillary 4 electrophoresis, data collection, and sequence analysis were carried out on an automated DNA sequencer (ABI 3700).

### Analysis of confirmation datasets microarrays

The microarray datasets (Affymetrix GeneChip Human Genome HG-U133B) performed by Metzeler KH et *al*. [6], Bullinger L *et al*. [7] and Gaidzik VI *et al*. [8] were downloaded from the Gene Expression Omnibus website (http://www.ncbi.nlm.nih.gov/geo/) with accession numbers GSE12417, GSE8653 and GSE 23312, respectively. The whole raw data were normalized using RMA (Robust Multiarray Averaging method) with the Expression Console software from Affymetrix. There were three probesets targeting *CXXC5* mRNA expression. Expression score (Normalized and log2 transformed) were reduced in one expression score by principal component analysis using the first principal component. The factor scores were also rescaled so that the minimal value of the score for each gene is 0 and the maximal value is 1.

### RINF silencing and chemosensitivity of hematopoietic AML cell lines

In vitro culture and lentiviral transduction of human AML cell lines. The K562 AML cell line was cultured in RPMI 1640 (Life Technologies) supplemented with 10% fetal bovine serum (Biochrom AG), 2 mM L-Glutamine, 50 units/ml penicillin G and 50 μg/ml streptomycin (Life Technologies). For *RINF* knock-down experiments, AML cells were transduced with pTRIP lentiviral vector that drives the constitutive expression of GFP (Green Fluorescent Protein) for cell sorting, and either a short-hairpin RNA targeting *RINF* sequence (shRNA-RINF) or an non relevant sequence (non-target-shRNA control).

WST-1 assay for assessment of cell proliferation/ viability. Cell viability was measured using the WST-1 assay (Roche Diagnostics, Paris, France). The myeloid cells were seeded (10^5^ cells/mL) in triplicates in 96-well plates (Costar, Cambridge, MA) and cultured for 24 h prior to addition of daunorubicin. After 48h of culture the WST-1 labeling mixture (10 μl) was added and the cells were incubated for additional 30-120 minutes. The absorbance of the samples against a background control of medium alone was measured at 450 nm.

### Apoptosis assay

For the cell lines apoptosis was assessed by TUNEL technology using “In Situ Cell Death Detection Kit, TMR red” (Roche Diagnostics, according to manufacturer's protocol), which label free 3'-OH DNA cleavage observed during apoptosis. Briefly, 2×10^6^ cells were washed with PBS solution and fixed using a 2% paraformaldehyde solution in PBS pH7.4 for 20 min. Cells were washed again once and permeabilized using freshly prepared 0.1%Triton −0.1% sodium citrate buffer for 8 min on ice. After 2 washes, cells were incubated for 1h in TUNEL reaction mixture containing Terminal deoxynucleotidyl transferase which catalyses polymerization of labeled nucleotides to free 3'OH-DNA ends of DNA strand breaks (mixed to label solution 1:50). The cells were finally washed three times, dropped on glass slides and mounted in Vectashield mounting medium containing 4-, 6-diaminidine-2-phenylindole (DAPI 6 Vector Laboratories) to counterstain nuclei. TMR red labeled cells (apoptotic cells) were detected by fluorescent microscopy (Leica DMRD, equipped with 63x objective, Leica, Wetzlar, Germany) and FACS analysis.

### *In vitro* culture of primary human AML cells

Cells were cultured in StemSpan serum-free medium supplemented with 100 μg/ml of gentamicin (Stem Cell Technologies Inc, Vancouver, BC, Canada) [9, 10]. Lenalidomide (Selleck Chemicals, Munich, Germany) was dissolved in DMSO and used at final concentrations of 0.05, 0.1, 0.5, 1.0, 5.0 and 10 μM. Pilot experiments demonstrated that DMSO at the final concentrations used in the experiments did not affect AML cells. Leukemic cells (1 × 10^6^ cells/ml) were cultured at 37°C in a humidified atmosphere of 5% CO_2_ for 24 hours before analysis by flow cytometry of AML cell viability/apoptosis (annexin expression/propidium iodide exclusion) and calreticulin surface exposure after live-dead gating [9, 10].

### Statistical analyses

Comparisons of the *RINF* mRNA expression levels in different subgroups of AML were performed using the Mann-Whitney rank test (for independent samples) and Wilcoxon's rank test (for dependent samples) by using the statistical software package SPSS 17.0. Spearman's test was used for correlation analyses. Survival data was analyzed using log-Rank test (Kaplan-Meier).

## Supplementary Figures and Tables




