# Supplementary material for: CXXC5 (Retinoid-Inducible Nuclear Factor, RINF) is a Potential Therapeutic Target in High-Risk Human Acute Myeloid Leukemia
Source: Oncotarget. 2013 Aug 15;4(9):1438–48. doi: 10.18632/oncotarget.1195 (PMC3824541; doi:10.18632/oncotarget.1195)
Supplement: Supplementary file 1 [file oncotarget-04-1438-s001.pdf]

CXXC5 (Retinoid-Inducible Nuclear Factor, RINF) is a Potential Therapeutic Target in High-Risk Human Acute Myeloid Leukemia – Astori et al

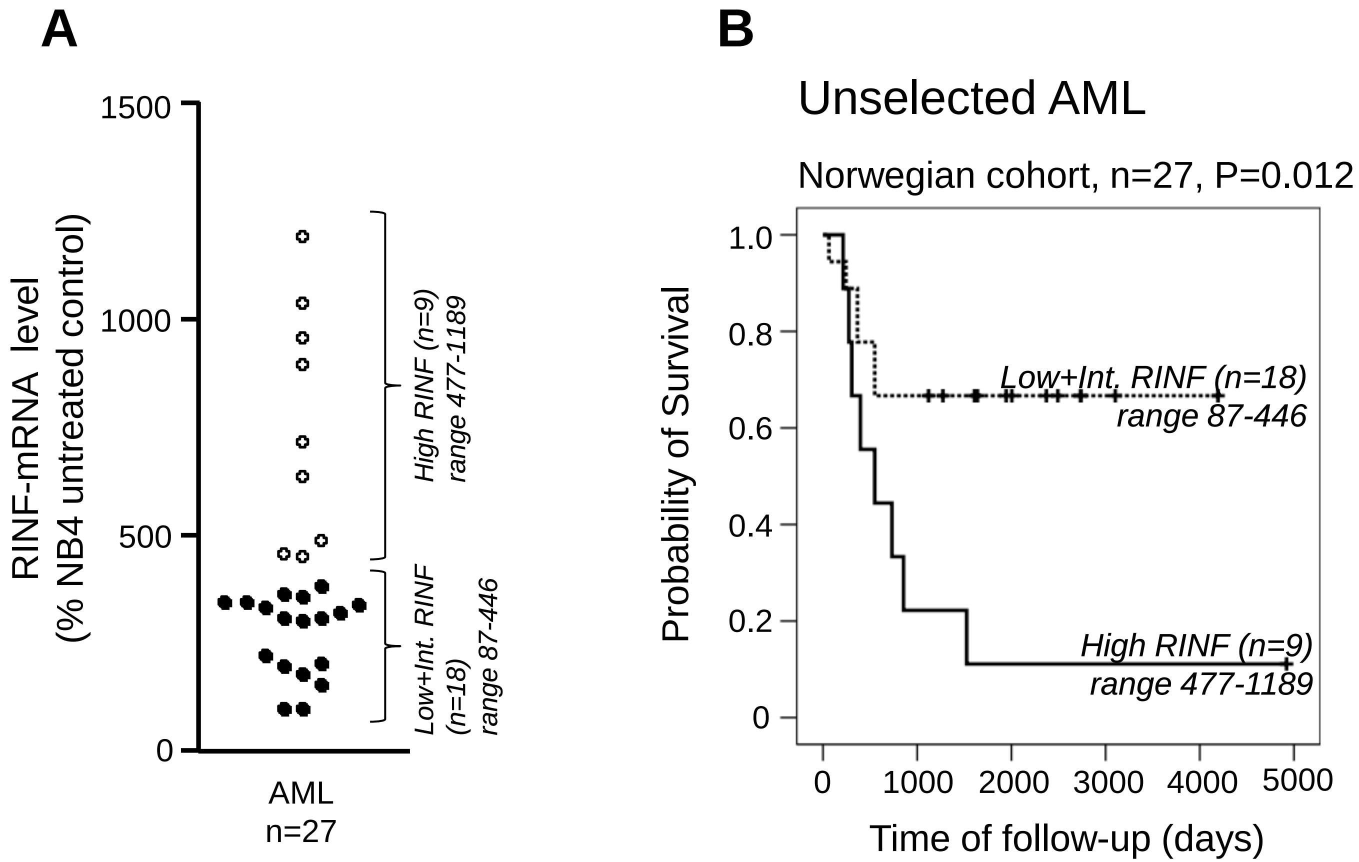

Supplementary Figure 1: The association between RINF expression and overall survival; analysis of the Norwegian patients with newly diagnosed AML and receiving intensive chemotherapy. Patients were classified as low, intermediate and high RINF expression. (A) The RINF expression level for the three groups. (B) Overall survival for the patients.

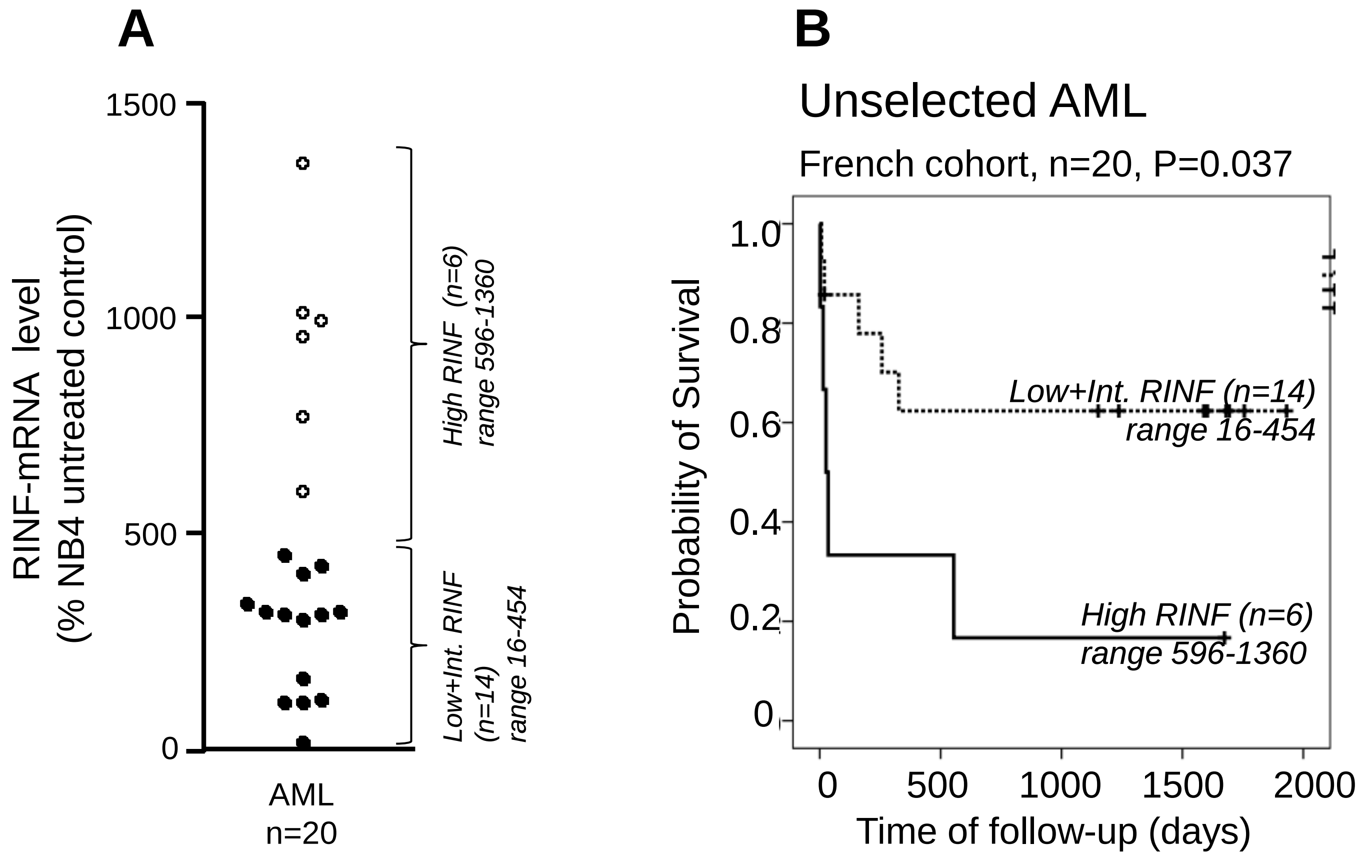

Supplementary Figure 2: The association between RINF expression and overall survival; analysis of the French patients with newly diagnosed AML and receiving chemotherapy. Patients were classified as low, intermediate and high RINF expression. (A) The RINF expression level for the three groups. (B) Overall survival for the patients.
