# Supplementary material for: CXXC5 (Retinoid-Inducible Nuclear Factor, RINF) is a Potential Therapeutic Target in High-Risk Human Acute Myeloid Leukemia
Source: Oncotarget. 2013 Aug 15;4(9):1438–48. doi: 10.18632/oncotarget.1195 (PMC3824541; doi:10.18632/oncotarget.1195)
Supplement: Supplementary file 2 [file oncotarget-04-1438-s002.pdf]

CXXC5 (Retinoid-Inducible Nuclear Factor, RINF) is a Potential Therapeutic Target in High-Risk Human Acute Myeloid Leukemia – Astori et al

Supplementary Table 1

Clinical and biological characteristics of the subset of Norwegian patients with newly diagnosed AML who received intensive chemotherapy (n=27).

Patient characteristics	Range	Mean / Median	Number of Patients
Age at diagnostic (years)	29-72	53 / 58	27
Gender (male/female)			17 / 10
**FAB classification**			**Number of Patients**
M0			1
M1			4
M2			6
M3			0
M4			8
M4e			1
M5			7
**Cytogenetics***			**Number of Patients**
Normal			15
Good			4
Intermediate			1
Adverse			6
Not determined			1
**Gene Mutations**			**Number of Patients**
FLT3-ITD-			16
FLT3-ITD+			10
Not determined			1
NPM1-			12
NPM1+			13
Not determined			2

* Cytogenetic abnormalities were classified according to the MRC guidelines and were available for 100 % of the patients. FLT3-ITD and NPM1 analyses were available for 26 (96%) and 25 (92%) patients, respectively. FAB classification was available for 100 % of the patients

Supplementary Table 2

Clinical and biological characteristics for the French AML patients receiving chemotherapy (n=20).

**Patient characteristics**	**Range**	**Mean / Median**	**Number of Patients**
Age at diagnostic (years)	21-87	67.3 / 72.5	20
Gender (male/female)			13 / 7

**FAB classification**	**Number of Patients**
M0	0
M1	7
M2	3
M3	0
M4	4
M4e	1
M5	5

**Cytogenetics***	**Number of Patients**
Normal	12
Good	1
Intermediate	5
Adverse	2

**Gene Mutations**	**Number of Patients**
FLT3-ITD-	13
FLT3-ITD+	7
NPM1-	12
NPM1+	8

* Cytogenetic abnormalities, FAB classification, FLT3-ITD and NPM1 analyses were available for all the patients.
